# Therapeutic Cancer Vaccines—T Cell Responses and Epigenetic Modulation

**DOI:** 10.3389/fimmu.2018.03109

**Published:** 2019-01-25

**Authors:** Apriliana E. R. Kartikasari, Monica D. Prakash, Momodou Cox, Kirsty Wilson, Jennifer C. Boer, Jennifer A. Cauchi, Magdalena Plebanski

**Affiliations:** ^1^Translational Immunology and Nanotechnology Unit, School of Health and Biomedical Sciences, RMIT University, Bundoora, VIC, Australia; ^2^Department of Immunology and Pathology, Monash University, Melbourne, VIC, Australia

**Keywords:** cancer vaccine-adjuvants, T cells, epigenetics, DNA methylation, histone modifications, microRNAs, long non-coding RNAs, biomarkers

## Abstract

There is great interest in developing efficient therapeutic cancer vaccines, as this type of therapy allows targeted killing of tumor cells as well as long-lasting immune protection. High levels of tumor-infiltrating CD8^+^ T cells are associated with better prognosis in many cancers, and it is expected that new generation vaccines will induce effective production of these cells. Epigenetic mechanisms can promote changes in host immune responses, as well as mediate immune evasion by cancer cells. Here, we focus on epigenetic modifications involved in both vaccine-adjuvant-generated T cell immunity and cancer immune escape mechanisms. We propose that vaccine-adjuvant systems may be utilized to induce beneficial epigenetic modifications and discuss how epigenetic interventions could improve vaccine-based therapies. Additionally, we speculate on how, given the unique nature of individual epigenetic landscapes, epigenetic mapping of cancer progression and specific subsequent immune responses, could be harnessed to tailor therapeutic vaccines to each patient.

## Introduction

To address the possibility of designing therapeutic cancer vaccines to work optimally in patients whose immune system may have been epigenetically modified, either by cancer cell-driven immunomodulation or by other external cues such as previous chemotherapy, it is first necessary to understand the different types of epigenetic imprinting that may be induced by vaccine therapy. Herein, we will firstly introduce fundamental concepts, and then review in depth: (1) the epigenetic mechanisms involved in vaccine-induced T cell mediated immunity, (2) T cell responses and epigenetic modulations induced by adjuvant systems to promote an anti-cancer environment, and (3) the epigenetic mechanisms involved in cancer immune escape, and possible ways to counteract them. On this basis, the potential use of the knowledge in epigenetic mechanisms to improve vaccine-based therapy will be discussed. Additionally, given epigenetics are both heritable and flexible following environmental cues (1), the epigenetic profile of each individual is unique. Based on this fact we also discuss the potential use of epigenetic biomarkers to diagnose cancer and predict an individual's immune response to therapeutic cancer vaccines.

### Vaccines Can Induce Effective Tumor-Specific T Cell-Mediated Immunity

Tremendous scientific advances have been made in the last decade in therapeutic cancer vaccine development, with many entering phase II and phase III clinical trials (2). Most cancer vaccines in development aim to promote tumor-associated antigens to be presented by antigen presenting cells (APCs) to generate long-lasting T cell immunity against cancer (3). Because dendritic cells (DCs) are the most efficient APCs, effective presentation of tumor antigens by DCs is considered a key determinant for cancer vaccine development (4).

Usually, the immune system identifies and destroys neoplastically-transformed cells. This immune surveillance mechanism functions as the body's primary defense against cancer. CD8^+^ T lymphocytes are the primary player in the recognition and destruction of cancer cells (5, 6). Following stimulation through tumor antigen recognition presented by DCs, naive CD8^+^ T cells are stimulated to proliferate and differentiate into effector cells, namely cytotoxic T lymphocytes (CTLs). Following recognition of major histocompatibility complex (MHC) class I-antigen complexes on tumor cell surface, activated CTLs induce tumor cell lysis by secreting perforin, granulysin and granzyme, as well as producing the death ligands including Fas Ligand (FasL) and tumor necrosis factor (TNF)-related apoptosis inducing ligand (TRAIL) (7). A subset of antigen-specific T cells will differentiate into memory cells for long-lived anti-tumor protection. DCs also activate CD4^+^ T helper (Th) cells, that are critical for CD8^+^ T cell activation (8). This cross-priming is required to produce effective and durable CTL responses by breaking cross-tolerance and providing protection of CTLs from activation-induced cell death (AICD) (8, 9). Additionally, Th cells are also capable of eradicating tumor cells following activation (10, 11).

**Figure 1:**
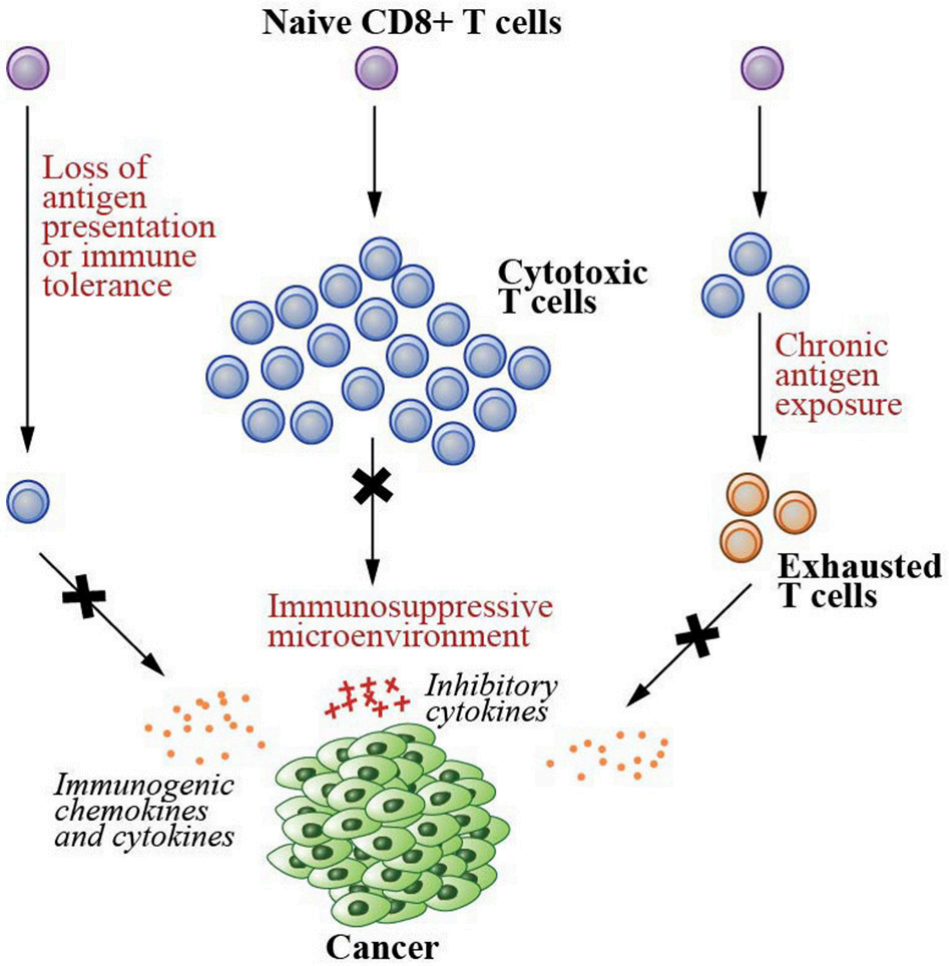
Failed immunity conditions that can be rescued by therapeutic cancer vaccines. Therapeutic cancer vaccines generate *de novo* T cell immunity that can repair the conditions that cause the failure of T cell-mediated immunity. These conditions include (1) having a low number of tumor specific T cells due to the lack of tumor antigen presentation and development of immune tolerance, (2) suppression of T cell infiltration into the solid tumor mass due to immunosuppressive microenvironments created by the cancer cells, and (3) T cell dysfunction/exhaustion due to chronic antigen exposure.

Several conditions, however, result in the failure of the immune system to destroy malignant cells (Figure 1). These include having a low number of tumor-specific T cells, suppression of T cell infiltration into tumor microenvironments, and T cell dysfunction/exhaustion (5, 6, 12–14). A low number of tumor-specific T cells results in a reduced number of cells capable of recognizing and killing neoplastic cells, hence tumor immune escape (6). Both failure in tumor antigen presentation and the development of immune tolerance contribute to this condition (5, 6). As tumor cells develop into a solid tumor mass, they create an immunosuppressive local microenvironment by secretion of specific factors that may restrict T cell infiltration, inactivate CTLs, and induce T cell apoptosis (13), further hampering cancer elimination. Due to chronic antigen exposure, T cells can also become dysfunctional and exhausted (12, 14). These T cells exhibit loss of the effector functions and upregulation of their immune checkpoint receptors such as PD1 and LAG3, the receptors that promote tolerance induction that subsequently prevents T cell activation upon stimulation.

To create neoplastic immunity, patients need to increase both the number and functionality of their cancer-specific T cells. This currently can be achieved by *de novo* generation of T cell-mediated immunity (15–18), through presentation by DCs (19, 20). One strategy utilizes a patient's own DCs as the therapeutic vaccine. DCs are maturated *ex vivo* using stimulatory cytokines and toll-like receptor (TLR) agonists, such as a combination of interferon (IFN)γ and lipopolysaccharide (LPS), and then loaded with patient-specific tumor antigens or proteins (21). The cells are then intradermally injected back into the patient together with adjuvants with the aim of generating a prolonged host immune response (22). In 2010, this strategy resulted in the first US Food and Drug Administration (FDA)-approved cancer vaccine, called Sipuleucel-T for prostate cancer patients (23). Increased survival in patients who received this personalized DC vaccine was achieved, suggesting successful long-lasting T cell immunity (24). Whilst this strategy has been successful in some patients, it has generally been inefficient. This is because the *ex vivo* DC vaccine preparation alters DC viability and functionality, is laborious and the output is of variable quality (19, 20). Moreover, the autologous DC generated from the patient's peripheral blood DC precursors, may have already been the subject of epigenetic imprinting by chemotherapy, radiation, immunotherapy or immune dysregulation by cancer cells, as such therapies have been shown to induce phenotypic alterations in immune cells (25). Understanding and modifying the epigenetic imprint of DC *ex vivo* (26), for example by the use of epigenetic modulators during tumor antigen loading, offers an intriguing avenue for future therapeutic exploration. Another strategy that currently holds promise in cancer vaccine development includes the injection of antigenic peptides or genetic material encoding for these peptides, in combination with adjuvants, to target DCs *in vivo*. However, despite appropriate antigen and adjuvant selections, many therapeutic cancer vaccines still fail to provide sustained T cell immunity, due to the many immune escape mechanisms available to neoplastic cells.

### Examining Epigenetic Involvement in T Cell Immunity Against Cancer

Recently several studies, as discussed in (27–31) show that epigenetic mechanisms drive phenotypic changes in both immune and cancer cells during their interactions. Epigenetics examines chemical modifications to a cell's deoxyribonucleic acid (DNA) that alters gene expression and thus the properties and behavior of cells, without changing their DNA sequence. These modifications include DNA methylation, histone modifications and ribonucleic acid (RNA)-associated mechanisms, via microRNAs (miRNAs) and long non-coding RNAs (lncRNAs) which mediate alterations in chromatin accessibility at regulatory regions that determine cell fate (32–35). For example, DNA methylation results in a closed conformation of the chromatin, inhibiting binding of the transcription machinery and thus preventing gene expression (32). Various histone modifications, on the other hand, regulate cellular gene expression by modifying the polarity of the nucleosome particle, and/or by recruiting protein complexes, to result in either a closed or open chromatin conformation (33). Similarly, lncRNAs regulate gene expression by direct binding to chromatin remodeling complexes and targeting them to specific genomic loci to alter DNA methylation or histone marks (35). Additionally, miRNAs are able to regulate gene expression post-transcriptionally (34). In the following section we will discuss epigenetic changes in both immune and cancer cells that may be induced by cancer vaccine therapy.

## Epigenetic Mechanisms Involved in Vaccine-Induced T Cell Immunity

### Epigenetic Mechanisms Involved in Vaccine-Induced CD8^+^ T Cell Differentiation Into Effector Cells

**Figure 2:**
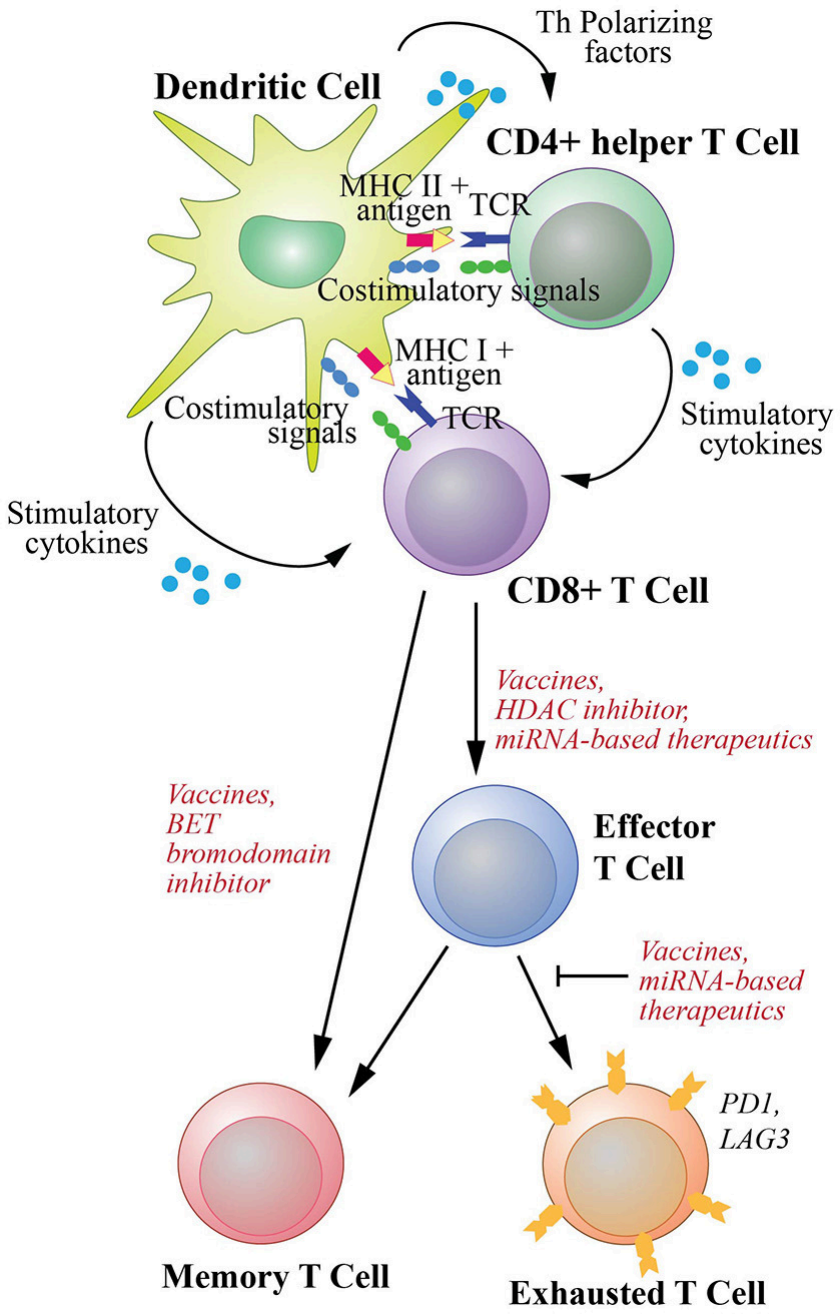
T cell activation and differentiation into effector cells and subsequent memory and exhaustion phenotypic changes. Differentiation of naive CD8^+^ T cells to cytotoxic T cells (CTLs) requires three signal interactions with dendritic cells (DCs). These include (1) the engagement of the T cell receptors (TCRs) with antigens as complexes with MHC class I molecules, (2) the interaction of DC costimulatory molecules with their receptors on CD8^+^ T cells, (3) stimulatory cytokines secreted by DCs to activate T cells. Additionally, co-stimulation of CD8^+^ T cells by T helper cells activated by DCs through MHC class II-antigen-TCR and costimulatory molecule complexes are required to promote efficient and durable CTL responses. The differentiation and activation of CD8^+^ T cells could potentially be enhanced by an HDAC inhibitor and miRNA-based therapeutics. Differentiation of naive cells into memory T cells is required for long-lasting protection and can be enhanced by a BET bromodomain inhibitor. Furthermore, upon chronic exposure to antigen, T cells can develop exhaustion phenotype. However, this exhaustion can be counteracted by cancer vaccines that generate *de novo* T cell immunity. miRNA-based therapeutics could potentially be used to help rejuvenate exhausted T cells.

Therapeutic cancer vaccines commonly utilize tumor-associated antigens presented by DCs to expand naive CD8^+^ T cells and drive their differentiation into both effector and memory cells. Activation of CTLs requires three signals (Figure 2): the first originates from the engagement of the T cell receptors (TCRs) with antigens as complexes with the MHC class I molecules on the surface of DCs; the second is the interactions of costimulatory molecules of DCs with cognate receptors of T cells including interactions between CD80/CD86 and CD28, CD70 and CD27, 41IBBL and 41IBB, OX40L and OX40, as well as GITRL and GITR (8, 36); and the third derives from cytokines including interleukin (IL)2 and IL12 secreted by DCs (37). Additionally, the tumor specific DCs activate Th cells through the interactions between TCRs and MHC class II-antigen complexes as well as the binding between their costimulatory molecules, such as the binding between CD80/CD86 and CD28. The activated Th cells in turn license DCs by upregulating their CD40L and LTαβ to interact with CD40 and LTαβR on DCs, respectively (36). The licensed DCs then produce polarizing factors such as IL12 to further differentiate CD4^+^ helper cells. The licensed DCs also increase the expression of CD80, CD70, OX40L, 41BBL, and GITRL, and secrete stimulatory cytokines such as IL2, IL12 and IFNγ, to generate CTLs with prolonged life-span with more effective effector function as reviewed in (8, 9, 36) (Figure 2).

Existing effector memory T cells can rapidly expand upon effective vaccination and differentiate into effector T cells to further mediate specific tumor destruction (15, 16). The vaccine-induced generation of antigen-specific T cells with distinct cellular phenotypes from genetically identical naive cells is mostly mediated by global epigenetic reprogramming. Recent work shows that epigenetic mechanisms control gene expression during CD8^+^ T cell differentiation following activation (27, 31). Epigenetic profiles also provide heritable maintenance of the phenotype of the differentiated T cells, following signal withdrawal (27, 31, 38, 39).

DNA methylation plays a significant role in CD8^+^ T cell differentiation into both effector and memory cells. In mammals, DNA methylation occurs mostly on CG dinucleotides (CpG). DNA methylation in CpG islands, short regions in the genome with high frequency of CpGs, is associated with transcriptional repression (32). During CD8^+^ differentiation, CpG islands become highly methylated at the promoters of silenced genes, and demethylated at the promoters of expressed genes (40–42). This alteration in methylation pattern dictates lineage-specific changes during differentiation following antigen-induced activation (43).

Like DNA methylation, promoters and other regulatory regions in the genome also undergo histone modifications during CD8^+^ T cell differentiation. Multiple studies show that in effector cells at the gene loci that are reduced in expression such as the memory cell-associated genes, activating histone marks including acetylation at lysine 9 on the histone 3 tail (H3K9Ac) and trimethylation at lysine 4 on the histone 3 tail (H3K4me3) are lost (41, 44–52). At the same gene loci, repressive marks including DNA methylation and trimethylation at lysine 27 on the histone 3 tail (H3K27me3) are gained. On the other hand, in the same cells, the effector cell-associated genes are upregulated and demonstrate decreased repressive and increased activating epigenetic marks (41, 44–52).

Importantly, in the absence of antigen presentation, memory cell subsets maintain their epigenetic patterns in order to retain their cellular phenotype (53). DNA methylation patterns of memory cells for example are preserved after antigen is withdrawn. This indicates involvement of epigenetic regulation in the maintenance of cellular phenotype to promote long-lasting vaccine-induced immunity. Similarly, di-acetylated histone H3 (diAcH3) is highly present in the expressed gene loci of activated effector CTLs, and this epigenetic mark remains present in the acquired memory cells (54). Additionally, several gene loci in naive and memory T cells remain poised in a resting state by the presence of bivalent epigenetic marks; the activating H3K4me3 and the repressive H3K27me3. This bivalency has been shown to be a crucial mechanism in regulating T cell faith, since following antigen stimulation, the activated gene loci are readily resolved into a monovalent H3K4me3 state subsequently allowing rapid differentiation into effector cells (45).

Recently, epigenetic enhancers have been shown to regulate CD8^+^ T cell differentiation in response to antigen presentation. The activation of the enhancers during differentiation was mapped based on genome-wide analysis of several epigenetic marks including H3K4me3, H3K27ac, H3K4me1 and the binding of histone acetyltransferase p300 (49, 55). These regions display striking epigenetic specificity in naive, effector, and memory T cells. Distinct transcription factors have also been shown to bind specifically to the enhancers of different subsets of CD8^+^ T cells (40). Similarly, chromatin accessibility profiles indicate unique regulatory regions in different CD8^+^ T cell subsets that also correspond to the expression of subset-specific genes (56, 57).

Furthermore, the levels of epigenetic modifier and transcription factor expression are distinct amongst T cell subsets. These may influence the capacity of T cells to react upon antigen stimulation. Indeed, the lack of DNA methyltransferase 3A (DNMT3A), a *de novo* methylating enzyme, promotes bias toward memory cell differentiation (58). Absence of the epigenetic modifier methyl-CpG-binding domain protein 2 (MBD2) causes impaired T cell differentiation into the effector phenotype (59). The epigenetic modifier BMI1, a reader of H3K27me3, and EZH2, a writer of H3K27me3 are both highly expressed upon T cell stimulation and differentiation into effector cells (60, 61). Histone deacetylases, SIRT1 (50) and HDAC7 (54) as well as BRD4, a reader of acetylated lysines (62) epigenetically repress gene expression and have been shown essential in directing differentiation of CD8^+^ T cells to gain their effector function.

In effector T cells, transcription factor PRDM1/Blimp1 (63), TBX21/Tbet (64, 65), and ID2 (66) are highly expressed to control CTL function via epigenetic regulations. PRDM1 for example, has been shown to recruit the repressive epigenetic modifier G9A and HDAC2 to both *IL2RA* and *CD27* loci, promoting differentiation of CD8^+^ T cells into effector cells (51). TBX21 is necessary to induce the expression of IFNγ, granzyme B, and perforin, by inducing rapid DNA demethylation and histone acetylation at the promoters of these gene loci (67–69). Furthermore, in both naive and memory cells, EOMES (65, 70), TCF1 (71), and FOXO1 (72–75) are highly expressed and have been shown to readily promote differentiation of these cells into CTLs, although their mode of action in regulating epigenetic changes in T cells remains unexplored.

### Epigenetic Modifications in T Cell Exhaustion

Another benefit of therapeutic cancer vaccines is their potential to revitalize exhausted T cells, by promoting *de novo* generation of T cell-mediated immunity (15–18). Exhausted T cells are a hallmark of cancer and the result of persistent antigen stimulation (76). They exhibit defective proliferation capacities, impaired stimulatory cytokine secretion, increased checkpoint receptor expression, and impaired effector functions (76). Recent studies show direct involvement of epigenetic mechanisms in T cell exhaustion. For example, compared to functional T cells, exhausted T cells exhibit reorganization of chromatin accessibility and activation of the exhaustion-specific enhancers (77, 78). Exhausted T cells also exhibit lower levels of diacetylated histone H3 (diAcH3) in comparison to functional T cells (79).

Both DNA methylating enzymes, DNMT1 and DNMT3B are upregulated in exhausted T cells (80), whilst DNMT3A has been demonstrated to functionally establish *de novo* exhaustion-specific DNA methylation patterns (81). Indeed, by blocking *de novo* DNA methylation, exhausted T cells retained their effector function (81). In exhausted T cells however, the expression levels of checkpoint/coinhibitory receptors, including PD1 and LAG3 were highly elevated (78, 82), which correlated with demethylation (83) and binding of transcription factor GATA3, BLIMP1, IRF4, BATF, and NFATc1 to the gene loci (51, 82, 84).

Additionally, lncRNAs including lncRNA-CD244 and lncRNA-Tim3 (85, 86) and miRNAs including miR-720, miR-31, miR-92a-3p, miR-21-5p, miR-16-5p, miR-126, and miR-182-5p (87–89) are capable of inducing exhaustion phenotypic changes by targeting specific pathways that impair T cell effector function. Therapies targeting these regulatory RNAs therefore may help restore T cell anti-tumor functions.

### Potential Epigenetic Interventions to Improve Vaccines

Therapeutic cancer vaccines are able to direct the proliferation and differentiation of naive and memory CD8^+^ T cells into CTLs through epigenetic modifications. As previously discussed, the involvement of epigenetic modifiers and transcription factors have been observed in directing T cell differentiation. This knowledge could potentially be used to improve the efficacy of therapeutic cancer vaccines.

For instance, BRD4 and SIRT1 are known to regulate differentiation of naive T cells into CTLs (50, 62). The absence of these two epigenetic modifiers promotes T cell differentiation into memory cells. Inhibition of these two epigenetic modifiers using the pharmacological inhibitor JQ1, results in the differentiation of naive CD8^+^ T cells into memory T cells that are long-lived, self-renewing and provide maintenance of acquired functional immunity, indicating that this pharmacological agent can be used to help create long-lasting immune response (62).

Another example is histone acetylation in Tbet-mediated IFNγ expression in CTLs. An HDAC inhibitor, trichostatin-A (TSA), can bypass the control of Tbet in inducing IFNγ expression (90). As IFNγ is critical for CTLs to exert their tumor killing activities, this pharmacological epigenetic modifier could potentially be used to enhance the efficacy of cancer vaccines.

Recently generation of CTLs was shown to depend on T cell receptor-mediated let-7 miRNAs downregulation. Decrease of let-7 miRNAs is necessary for the acquisition of effector function through derepression of the let-7 targets (91). On the other hand, miR-155 is necessary to generate effector CD8^+^ T cells (92). Therefore, it has been suggested since that modulation of let-7 miRNAs or miR-155 can be used to potentiate immunotherapies for cancer.

Furthermore, as previously mentioned, therapeutic vaccines can reverse systemic exhaustion by promoting *de novo* generation of functional T cells. This T cell exhaustion phenomenon is dependent on the host DNA methylation profile. Recently, in mice, T cell exhaustion was successfully reversed by inhibition of *de novo* methylation using Decitabine, an FDA-approved DNA demethylating agent (81). Moreover, as exhausted T cells overexpress checkpoint receptors that prevent them from killing tumor cells, the use of checkpoint inhibitors has proven useful to remove such molecular breaks. Thus, these pharmacological agents could potentially be used in combination with therapeutic cancer vaccines to rejuvenate exhausted T cells, whilst effectively promoting new T cell-mediated immunity.

The magnitude of T cell activation and the accompanying epigenetic modulations dictate the efficacy of a vaccine being developed. The strength of the immune response elicited by vaccines is also highly dependent on the chosen antigens. Several strategies have been recently implemented to optimize this selection. These include personalized peptide vaccines that utilize multiple cancer peptides to complement pre existing host immunity (93). Another strategy is using neoantigens, that is, antigens that arise because of mutations in tumor cell DNA. Once identified, patient's T cells are used to screen which neoantigens harness the potential for effective antitumor responses. Vaccines are then developed based on these screening results. Recently, cancer-specific epigenetic marks have been explored to be used as therapeutic target antigens in vaccines. For instance, several miRNAs have been used in cancer vaccine development (94). Such strategies may provide significant additional resources for individualized cancer treatment.

## T Cell Responses and Epigenetic Modulations Induced by Adjuvant Systems to Promote an Anti-cancer Environment

Adjuvants have long been an integral component of vaccines to elicit a strong antigen-specific T cell-mediated immune response. Classically, adjuvants allow gradual antigen release or increase antigen recognition by innate cells to create a prolonged immune response elicited by the vaccine. Alternatively, delivery systems may be used to efficiently deliver a specific antigen to APCs. Nowadays, adjuvants in therapeutic cancer vaccines are not only used to improve anti-tumor immunity, but they are also selected based on their properties that directly promote tumor cell killing and induce an anti-tumor microenvironment. Additionally, adjuvants and delivery systems that promote CD8^+^ T cells are optimal for cancer vaccine development, though historically many adjuvants have been poor inducers of a CD8^+^ T cell response. Here, we describe key adjuvants and delivery systems that have progressed to investigation in human clinical trials in cancer patients. Subsequently, we discuss the epigenetic modulations induced by adjuvants, and how such modifications may facilitate vaccine-based therapies in cancer patients.

### Vaccine Adjuvants

In most cancer vaccines, adjuvants and immunostimulants are chosen to facilitate generation of CD8^+^ T cell responses to MHC class I-presented tumor antigens. For this reason, the adjuvant should activate APCs such as DCs, promote antigen presentation and subsequent presentation to induce secretion of stimulatory cytokines, such as IFNγ, IL12, and IL2 (Figure 2). Adjuvants that promote cytokine production and Th1 differentiation (95) are desired as Th1 cells costimulate native CD8^+^ T cells to differentiate into CTLs (8) (Figure 2). Moreover, following the stimulation, Th1 cells produce IFNγ that in turn increase antigen presentation on cancer cells (10), to enhance direct killing of tumor cells (11) as well as create an immunogenic tumor microenvironment (96), thus further helping tumor control. Adjuvants additionally can be selected based on their ability to induce specific innate cells such as natural killer (NK) cell-mediated tumor killing. NK cells are the effector cells of the innate immune system that upon stimulation can directly lyse tumor cells via perforin and granzyme (7). They also have a main role as rapid and potent cytokine producing cells, such as IFNγ and TNFα, that stimulate killing through the death receptor pathways (7, 96). Moreover, NK cells induce DC maturation and amplify T cell anti-tumor responses (97).

One of the main antigen recognition and activation pathways utilized by APCs are TLRs. TLRs are receptors expressed by APCs that can recognize conserved structures derived from pathogens, namely MAMPs (microbe-associated molecular patterns). The same receptors can also recognize DAMPs (damage-associated molecular patterns) that are expressed by cells under conditions of stress. TLR ligands/agonists are widely used to stimulate innate immune responses. TLR agonists, especially those targeting endosomal TLRs, have been shown to generate anti-tumor immunity (98). Thus, cancer vaccines targeting TLR activation could result in the generation of a range of cytokines that stimulate a Th1 bias, as well as promote CTL induction and NK cell-mediated killing that can then be utilized for directed tumor treatment strategies (99).

TLR3, TLR7, TLR8, and TLR9 are predominantly endosomal. It is known that different subsets of DCs have been shown to express distinct arrays of TLRs (100). TLR3 for example is predominantly expressed in conventional DCs (101). This subset of DCs are especially efficient in activating CD8^+^ T cells and inducing adaptive immune responses against tumor cells (100). Additionally, several cancer cells have been shown to express TLR3 at various levels, including hepatocellular carcinoma (102), breast cancer (103), and neuroblastoma (104). Polyinosinic:polycytidylic acid (Poly I:C) and polyadenylic:polyuridylic acid (Poly A:U) are synthetic analogs of viral dsRNAs which are recognized by TLR3 (105) that have been extensively used as an adjuvant in many clinical trials for cancer vaccines (106). The agonists of TLR3 are capable of activating APCs and cancer cells to induce secretion of inflammatory cytokines including type1 interferons that in turn activate T cell responses against cancer cells (107, 108). Poly I:C is also capable of reversing the pro-cancer innate immune response to anti-cancer immunity, especially within the tumor microenvironment (109). In clinical trials, albeit with limited numbers of patients, both Poly ICLC and Poly I:C12C, modified versions of Poly I:C, were shown to boost anti-tumor activity by inducing potent tumor-specific CTL and NK responses (110, 111).

TLR8 is expressed by conventional DCs and monocytes, whereas TLR7 is expressed predominantly in plasmacytoid DCs (101). Plasmacytoid DCs are a major producer of stimulatory cytokines in response to many viral infections (100). The ligands of TLR7 and TLR8 have been exploited as adjuvants. Their receptors are similar in structure but promote secretion of distinct sets of proinflammatory cytokines by APCs. TLR7 induces the secretion of type I interferons such as IFNα, while TLR8 promotes secretion of TNFα and IL12 (112). Both receptors are endosomal and recognize viral ssRNA (105) and also bind their synthetic analogs, including imiquimod and resiquimod (113, 114). In clinical studies, TLR7/8 agonists enhanced CD8^+^ T cell responses of a vaccine to prostate-specific peptide and NY-ESO-1, an tumor-specific antigen (115). Additionally, imiquimod has been approved for the treatment of basal cell carcinoma by the FDA (116).

TLR9 agonists are also potent adjuvants. TLR9 itself is predominantly endosomal, and present abundantly in DCs, especially plasmacytoid DCs. It binds microbial DNA, recognizing in particular the unmethylated CpG motifs in viral and bacterial genomes (105). The synthetic TLR9 ligand, CpG oligodeoxynucleotide (CpG-ODN), a short unmethylated ssDNA, activates DCs to secrete type I interferons, and promotes a strong CTL response (117). When used in combination with DC-based cancer vaccines, CpG-ODN enhances CD8^+^ T cell activity. In combination with tumor-specific-peptide-based vaccines, such as NY-ESO-1 and MART1, CpG-ODN resulted in elevated CD8^+^ T cell responses, however tumor eradication was rarely achieved (115).

Unlike their endosomal counterpars, TLRs expressed on the cell surface typically recognize extracellular foreign microbes. TLR4, one of the surface TLRs, recognizes LPS molecules of gram-negative bacteria (105). In humans, LPS can cause septic shock syndrome, due to its potent immune stimulatory activity (118). A derivative of LPS, monophosphoryl lipid A (MPL) in combination with the classical adjuvant alum, was licensed by the FDA for use as part of the human papillomavirus vaccine in 2009 (119). In clinical trials, MPL has also been used as an adjuvant for cancer vaccines to promote Th1-specific immune responses (120, 121).

Other adjuvants that have been used to induce T cell responses have included classic formulations/emulsions including oil or saponin. QS-21 is a potent saponin-based adjuvant that is isolated from *Quillaja Saponaria* (122). Although its mechanism of action is largely unknown, QS-21 has been shown to activate the secretion of IL2 and IFNγ, stimulate the proliferation of CTLs and induce Th1 bias (123). Formulations of QS-21 has been tested in human clinical trials for various cancer vaccines (124, 125). Another strong adjuvant that has been trialed for cancer vaccines is Montanide. The aim of this classical adjuvants is to allow sustained antigen release from the immunization site. This strategy is used to create a prolonged and higher amplitude of CTL-mediated immune response. Montanide-based adjuvants are water-in-oil emulsions that promote slow release of antigens and thus prolong antigen presentation to the immune system (126). In clinical trials, montanide ISA720 and ISA51 promote Th1 immune responses and significant CTL activation (127, 128).

### Delivery Systems

Several delivery systems, including virosomes, liposomes, viral-like proteins (VLPs), and immune-stimulating complexes (ISCOMs) have been developed and used in clinical trials to improve the efficacy of cancer vaccines. Virosomes are empty viral particles that can carry tumor-specific antigens as vaccines (129). In metastatic breast cancer patients, the modified influenza virosomes containing the breast cancer peptide (Her/neu^+^) are well tolerated and not only promote secretion of proinflammatory cytokines, including IL2, TNFα and IFNγ but also promote T cell immunity (130). Liposomes are synthetic phospholipid vesicles that work similarly to virosomes. They are often used to deliver messenger RNA (mRNA) encoding for a specific antigen (131). They have shown promise in delivering mRNA to APCs in clinical trials for non-small cell lung cancer, prostate cancer and follicular lymphoma patients (132, 133), and inducing antigen-specific CD8^+^ T cell responses. Liposomes that carry DNA have also been developed to stimulate TLR9, activate DCs and subsequently CTLs (134). VLPs are multimeric structures of viral proteins devoid of viral genetic material. Similar to native viruses, specific epitopes on VLPs can be recognized and presented by APCs to promote immune responses as reviewed in Ong et al. (135). VLPs have been developed for use in vaccines for various forms of cancer, including liver, cervix, lung, skin, breast, and prostate, as they not only promote antigen-specific immunity, but also counteract the immunosuppressive microenvironment created by a tumor mass (135). Finally, ISCOMs are composed of saponin, cholesterol and phospholipid. They are regularly used as a vaccine delivery system, however saponin can also stimulate the immune system by activating DCs and inducing robust antigen-specific T cell responses (136). Furthermore, ISCOMATRIX® has been used with the recombinant NY-ESO-1 protein in cancer patients to induce T cell immune responses (137, 138), however this vaccine failed to promote an adequate immune response in advanced melanoma patients (139).

### Epigenetic Modulations Induced by Adjuvants and Their Potential to Improve Cancer Vaccines

Whereas a number of whole-pathogen-based vaccines against infectious diseases have been shown to modulate the epigenetic landscape of immune cells, much less is known about the adjuvants and carriers used in cancer vaccines and patients. For example, vaccination with the bacillus Calmette-Guérin (BCG) vaccine for tuberculosis, has been shown to specifically alter epigenetic profiles of monocytes and broadly enhance protection against multiple infectious pathogens (beyond tuberculosis) in humans (140). This suggests that vaccines could leave stable epigenetic marks in certain immune cell populations and alter how the immune system reacts toward subsequent diverse challenges after vaccination, perhaps including cancer. In fact, the non-specific beneficial effects of the BCG vaccine are used in the clinic to treat bladder cancer (141). Modulation of T cell immunity using vaccines in combination with specific adjuvants may provoke changes in epigenetic profiles of immune cells and improve anti-tumor immunity. These beneficial epigenetic profiles may be further potentiated by the use of epigenetic modulating drugs. Indeed, epigenetic potentiation of the NY-ESO-1 protein vaccine with montanide-based adjuvant using decitabine, a DNMT inhibitor, has been successful in treating epithelial ovarian cancer (28).

Several adjuvants currently used in cancer vaccines are indeed capable of altering immune cell interactions with cancer cells by inducing stable epigenetic modifications in both host immune and cancer cells. These adjuvants could therefore be harnessed as promising candidates to promote beneficial epigenetic modulations in vaccine-based therapies. The use of TLR-ligand adjuvants could indeed be promising, as studies have shown that epigenetic reprogramming can be achieved via TLR stimulation. For instance, stimulating TLR3 with Poly I:C activates the epigenetic machinery causing a global change in the expression of epigenetic modifiers that in turn promotes chromatin remodeling and nuclear reprogramming (142). In addition, TLR3 receptor combined with Poly I:C directly promoted global DNA methylation in peripheral blood mononuclear cells (PBMCs) in pigs (143). Poly I:C promotes the expression of pro-inflammatory cytokines IL23 and IL33 by direct modification of the epigenetic marks on the promoters of these gene loci (144, 145). Furthermore, it reactivates the expression of several silenced miRNAs in tumor cells that subsequently leads to its direct anti-tumor activity (146). Such direct epigenetic modifications by Poly I:C are highly beneficial to improve the efficacy of therapeutic cancer vaccines.

Another advantageous cancer vaccine adjuvant candidate could be CpG-ODN, a ligand for TLR-9. Although there is less data available regarding the effects of CpG-ODN on global epigenetic reprogramming, it has been shown to promote chromatin changes in specific gene loci. For example CpG DNA induced production of IL12 due to its ability to elicit epigenetic modifications on the IL12p40 promotor, including histone acetylation and nucleosomal remodeling, which leads to gene activation (147). In cancer cells, CpG-ODN has been shown to directly exert its anticancer potential. For instance, in chronic lymphocytic leukemia (CLL), CpG-ODN promotes epigenetic changes associated with active transcription, namely, H3K9/K14 acetylation and H3K4 trimethylation at the promoter of *PRDM1* (148). PRDM1 expression promotes terminal differentiation of CLLs (149, 150), which is established as a potent therapy for CLL.

Epigenetic-modulating activites of TLR4 ligand adjuvants may mimic those exerted by LPS. This classical TLR4 ligand promotes innate immune responses by reprogramming monocytes to accumulate active histone marks such as H4Ac, at promoters of genes involved in inflammation and phagocytic pathways (151). However, further stimulation of innate immune cells by LPS can promote tolerance, by removal of H4Ac at promoters of inflammatory gene loci, such as *IL6 and TNF*α (152, 153). It was further identified that Trichostatin A, a deacetylase inhibitor could reverse the repression of *IL6* and restore H4Ac (152).

Additionally, adjuvants that deliver genetic materials can also potentially be used to promote beneficial epigenetic modulations during vaccine-based cancer therapies. RNA-LPX, a liposome-based adjuvant for example, has been shown to efficiently target DCs and promote strong antigen-specific T cell responses in melanoma patients (131). Since the expression of many miRNAs are altered in various cancer cells, such form of adjuvant could potentially be used to target miRNAs to both alter epigenetic imprinting in the cancer cells and promote cancer elimination.

Despite progression in the knowledge of adjuvants for cancer therapy, their mode of action and the precise epigenetic mechanisms involved are still largely unmapped. As discussed earlier, all types of adjuvants may exert direct and indirect effects, which might result in epigenetic modifications in the cells of the immune system and the associated cancer cells. The accumulating evidence highlighted above provides a rationale to investigate more broadly the potential use of epigenetic modifications by vaccine-adjuvants in the context of cancer therapy.

## Epigenetic Mechanisms Involved in Cancer Immune Escape and Ways to Counteract Such Mechanisms

**Figure 3:**
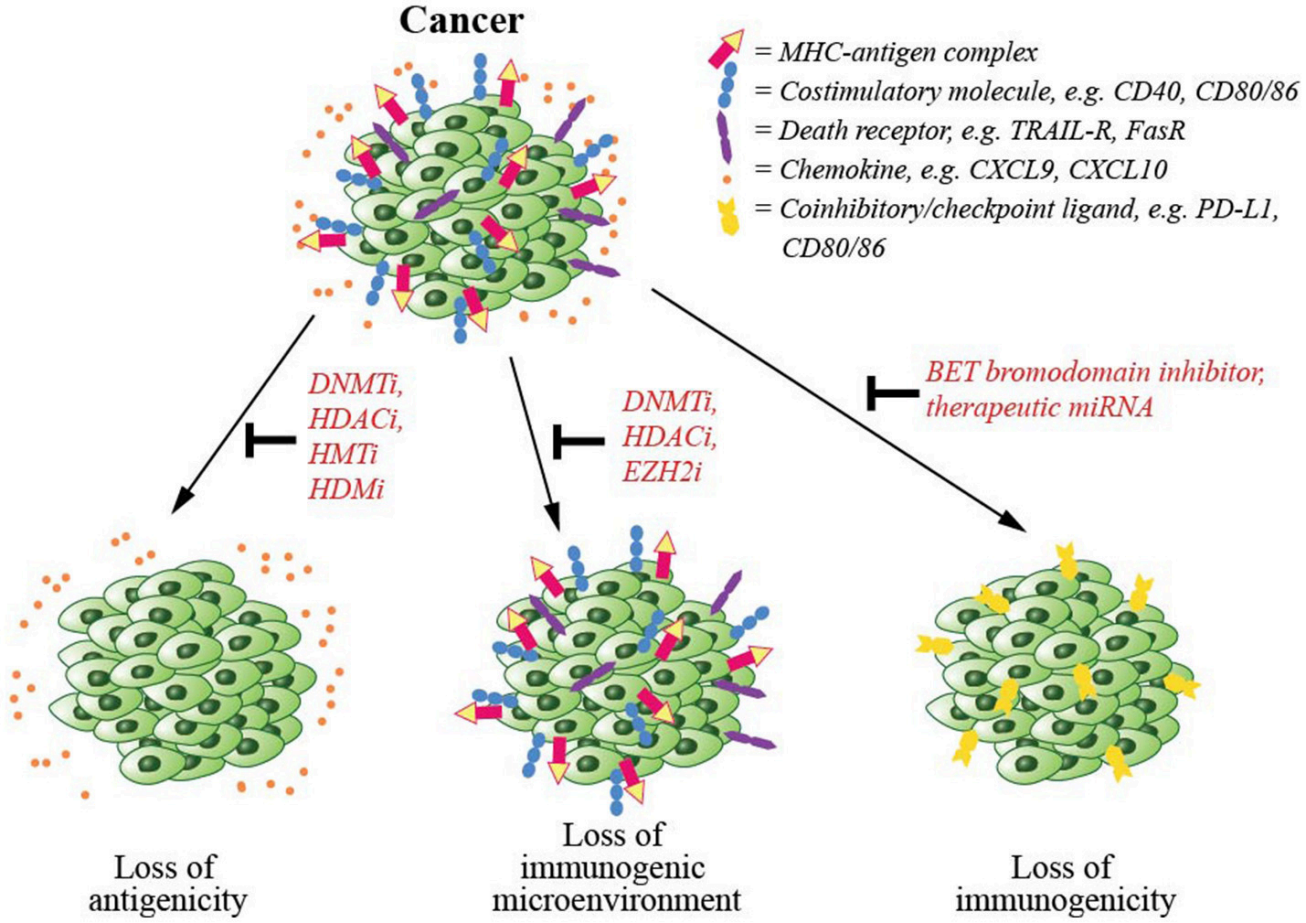
Cancer immune evasion and the epigenetic modifications counteracting such mechanisms. To escape from immune-mediated killing, cancer cells exploit several evasion strategies. These are (1) downregulation of antigen presentation and costimulatory molecules, which could be counteracted by the inhibitors of epigenetic regulators including DNA methyltransferase (DNMT), histone deacetylase (HDAC), histone acetyltransferase (HAT), or histone demethylase (HDM), (2) downregulation of chemokines that signal T cells to infiltrate tumor mass, again that could be inhibited by inhibitors of DNMT, HDAC, or EZH2, and (3) upregulation of coinhibitory/checkpoint ligands, which could also be blocked by a BET bromodomain inhibitor.

Disruption of epigenetic regulatory mechanisms is prevalent in cancerous cells leading to altered gene expression, perturbed functionality and malignant transformation. Due to the reversible nature of epigenetic modifications and their involvement in cancer, several epigenetic-modifying drugs have now been approved by the FDA for cancer treatment (154). Several mechanisms including downregulation of antigen presentation machinery, upregulation of coinhibitory/checkpoint ligands and establishment of a pro-cancer environment are involved in immune evasion by cancer cells (Figure 3). In this section, we will discuss epigenetic mechanisms involved in cancer escape from T cell-mediated immunity, and epigenetic drugs that may be able to counteract such mechanisms.

To escape from CTL-mediated killing, cancer cells commonly downregulate the expression of their antigens. This is achieved by epigenetically modifying their DNA, through methylation, commonly observed for MHC class I antigens, and via histone deacetylation, often seen for MHC class II antigens (155, 156). *In vitro*, the HDAC inhibitor mocetinostat increases antigen presentation by MHC class II molecules (157). Other available epigenetic drugs that may modulate the level of expression of antigens in cancer cells include histone methyltransferase (HMT) and demethylase (HDM) inhibitors (158, 159) (Figure 3). In patients, reduced expression of antigens and the components of antigen presentation machinery, such as MHC class I molecule has been shown to correlate with malignancy (160–162). Epigenetic-modifying drugs, such as DNMT and HDAC inhibitors have been widely used to reverse this downregulation of tumor antigens (154). DNMT inhibitors for example, including 5-azacytidine (5-AC) and 5-aza-2'-deoxycytidine (DAC) have been approved by the FDA for the pre-leukemic disorder myelodysplasia (MDS) (163).

The components of the cellular antigen presentation machinery including MHC class I, TAP1, TAP2, LMP2, and LMP7 are epigenetically downregulated in many cancers (164–166). Similarly, tumor cell downregulation of costimulatory molecules including CD40, CD80, CD86, and ICAM1 have been observed and associated with the rapid progression of various cancers as reviewed in (167). Additionally, cancer cell escapes from CTL-induced apoptosis by downregulating the expression of their death receptors, such as TRAIL-R and Fas (168). In *in vitro* and animal models, both DNMT and HDAC inhibitors have been shown to induce the expression of the antigen presentation molecules (164–166), surface costimulatory molecules and death receptors (166, 169–174), which then increases the sensitivity of tumor cells to immune-mediated cell killing.

Another known mechanism of immune evasion by cancer cells is to increase their expression of checkpoint ligands, such as PD-L1, CD80, and CD86 (Figure 3) and promote T cell tolerance. The use of DNMT and HDAC inhibitors in such cancer cells may synergistically upregulate the expression of the checkpoint ligands on the surface of cancer cells (175). This is however argued to be beneficial since these epigenetic-modifying agents will sensitize tumor cells for checkpoint inhibitor therapy and allow CTL-mediated killing (176, 177). On the other hand, a BET bromodomain inhibitor (JQ1), has been shown to directly downregulate the expression of checkpoint receptors on cancer cells, rendering them sensitive to CTL-mediated cell death (178, 179).

Many cancer cells suppress certain miRNA expression, in order to increase the expression of checkpoint ligands on their cell surface. These miRNAs include miR-34 (180), miR-29 (181), and miR-200 (182) in lung cancers, miR-138 in glioma (183), miR-187 in renal cell carcinoma (184) and miR424(322) in ovarian carcinoma (185). Based on this knowledge, therapeutic miRNAs could be developed to repress checkpoint ligand expression on the surface of cancer cells. However, their use as therapeutic treatment agents will require rigorous clinical testing as miRNAs may not be specific and thus pose significant concerns regarding non-specific adverse effects in patients.

Another recently identified mechanism of tumor immune escape is the repression of chemokine expression. Chemokines are required for T cell infiltration into the tumor microenvironment (Figure 3). For example, in ovarian cancer, tumor cell production of chemokines CXCL9 and CXCL10 are epigenetically repressed by EZH2-mediated H3K27me3 and DNMT1-mediated DNA methylation (186). Inhibition of EZH2 methytransferase increases chemokine production and improves T cell infiltration in patients with ovarian cancers (186). Similarly HDAC inhibitors have been used to increase chemokine expression and T cell infiltration in lung cancer patients (187).

Although epigenetic drugs are mainly used to target cancer cells, they may also exert their effects on host immune cells. For example, HDAC inhibitors can increase histone acetylation on several gene promoters in NK cells, including the death-induced receptors Fas and TRAIL-R2, which potentiate NK cell-mediated immune surveillance against cancer cells (173, 174, 188, 189). However, the global modulating effects of these drugs on T cells and other cells than cancer, are currently unknown.

Extensive clinical research has been carried out that has resulted in FDA approval for the use of seven epigenetic drugs for cancer treatment (154), though the role of such epigenetic inhibitors or modulators in altering the epigenetic landscapes of cells other than cancer cells is currently largely unknown. This is an important issue since epigenetic-modifying drugs as well as miRNA therapies, may not be specific, and thus may cause multiple unknown effects in patients. However, further clinical studies are certainly warranted to fully investigate potential treatment side-effects, especially when the epigenetic therapy is used in combination with immunotherapy, such as in cancer vaccines.

## Epigenetics as Cancer Biomarkers in Vaccine Immunotherapy

Epigenetic marks including DNA methylation, histone modification, and RNA-associated mechanisms, such as miRNAs and lncRNAs are found to be heritable mitotically from cell to cell and meiotically from generation to generation. Epigenetics has explained how gene activity can be modulated by external environmental factors, such as lifestyle and diet. Due to this unique characteristic, epigenetic marks gained from external environmental cues that shape the parent's DNA are heritable, thus allowing the transfer of experiences from the parents to offspring (190). A person's own lifestyle also shapes that individual's epigenetic profiles. As these profiles dictate cell identity and function, they also dictate individual susceptibility to diseases including cancer (191) and the capacity of their immune system to respond to different challenges. Such profiles can thus be exploited as non-invasive markers for cancer susceptibility, diagnosis and prognosis (192) and possibly predicting the effectiveness of vaccine therapy.

Epigenetic alterations can be readily detected as circulating biomarkers and may prove useful in clinical cases where surgery is contraindicated and biopsy results are inconclusive, such as in gliomas (193). Many circulating epigenetic biomarkers have been developed based on specific DNA methylation pattern of the cancerous cells, as reviewed in (194, 195). For example, in prostate cancer, methylated *MCAM* detects early stage of cancers with 66% sensitivity and 73% specificity, which is an improvement from PSA with only 42.8% sensitivity and 41.1% specificity (196). Circulating nucleosomes and histone modifications may also serve as markers to increase specificity and sensitivity of current diagnostic and prognostic tests as reviewed in (197, 198). Other attractive circulating epigenetic biomarkers in cancer are the circulating miRNAs, as reviewed in (199). For example, in pancreatic ductal carcinoma, miR-155-5p in plasma is a marker for cancer presence, and increased expression levels in cancerous tissues are associated with a more advanced tumor stage and poorer prognosis (200–202).

Importantly, such non-invasive biomarkers would also be effective tools for both choosing and monitoring the effectiveness of cancer vaccines for each individual case. For example, in gastric cancer, an increased plasma miR-222 level is significantly correlated with a more advanced tumor stage and a lower overall survival (203). This marker can thus be used to predict the outcome of the disease and in combination with T cell functional markers such as IL2, TNFα, and IFNγ could predict patient's response to specific cancer vaccine. Certainly, epigenetic marks identified in a person's immune cells, such as the levels of specific miRNAs involved in T cell effector function and T cell exhaustion, may be used as functional biomarkers to predict T cell activity following vaccine therapy and additionally to help create an effective combination therapy for that particular person.

## The Future of Therapeutic Cancer Vaccines as Immunotherapy

As therapeutic cancer vaccines evolve and additional knowledge of their mode of action is established, more effective personalized treatment strategies will be developed. Combination therapies for cancer using complementary vaccine-based therapy with epigenetic inhibitors and/or checkpoint inhibitors will also become more widely used. As the nature of both cancer cell and the associated host immune response is dependent on host epigenetic profiles, additional detailed knowledge of the epigenetic modulations involved in vaccine-generated T cell immunity against cancer cells could prove instrumental to the development of effective vaccine-based immunotherapy. Whilst the epigenetic landscape of cells is unique amongst individuals, specific epigenetic profiles of cancerous cells, as well as of immune cells may be harnessed as biomarkers for early detection of tumors, and also to guide the selection of a targeted therapy.

## Author Contributions

AK and MP contributed to the conception and design of the review study. AK wrote the first draft of the manuscript. AK, MDP, MC, KW, JB, JC and MP wrote sections of the manuscript. All authors contributed to manuscript revision, read and approved the submitted version.

### Conflict of Interest Statement

The authors declare that the research was conducted in the absence of any commercial or financial relationships that could be construed as a potential conflict of interest.
